# Limits of Spatial Resolution for Thermography and Other Non-destructive Imaging Methods Based on Diffusion Waves

**DOI:** 10.1007/s10765-013-1513-0

**Published:** 2013-10-02

**Authors:** Peter Burgholzer, Günther Hendorfer

**Affiliations:** 1 Christian Doppler Laboratory for Photoacoustic Imaging and Laser Ultrasonics, Research Center for Non Destructive Testing GmbH (RECENDT), Altenberger Strasse 69, 4040 Linz, Austria; 2 FHOOE Forschungs & Entwicklungs GmbH, Stelzhamerstr. 23, 4600 Wels, Austria

**Keywords:** Diffusion, Entropy, Fluctuation, Information, Non-destructive imaging, Resolution

## Abstract

In this work the measured variable, such as temperature, is a random variable showing fluctuations. The loss of information caused by diffusion waves in non-destructive testing can be described by stochastic processes. In non-destructive imaging, the information about the spatial pattern of a samples interior has to be transferred to the sample surface by certain waves, e.g., thermal waves. At the sample surface these waves can be detected and the interior structure is reconstructed from the measured signals. The amount of information about the interior of the sample, which can be gained from the detected waves on the sample surface, is essentially influenced by the propagation from its excitation to the surface. Diffusion causes entropy production and information loss for the propagating waves. Mandelis has developed a unifying framework for treating diverse diffusion-related periodic phenomena under the global mathematical label of diffusion-wave fields, such as thermal waves. Thermography uses the time-dependent diffusion of heat (either pulsed or modulated periodically) which goes along with entropy production and a loss of information. Several attempts have been made to compensate for this diffusive effect to get a higher resolution for the reconstructed images of the samples interior. In this work it is shown that fluctuations limit this compensation. Therefore, the spatial resolution for non-destructive imaging at a certain depth is also limited by theory.

## Introduction

**Fig. 1:**
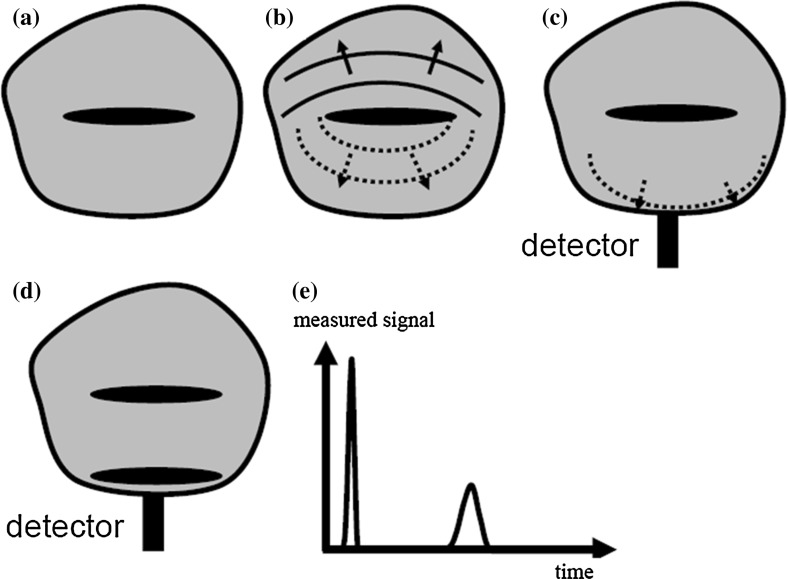
Information loss in non-destructive imaging: the spatial resolution is essentially influenced by the propagation of certain waves, e.g., thermal or ultrasound waves to the surface. Scattering, dissipation, or diffusion causes entropy production and a loss of information of the propagating waves: (a) Sample containing internal structure, which should be imaged. (b) Propagation of the waves to the sample surface: entropy production goes along with fluctuations (“fluctuation–dissipation theorem”) and causes a loss of information. (c) Detection of wave at the sample surface. (d) The same structure is contained twice in the sample: just beneath the surface and at a higher depth. (e) Measured signal at the detector surface as a function of time for the sample shown in (d): due to diffusion or dissipation, the signal from the deeper structure has not only smaller amplitude but it is also broadened compared to the signal from the structure just beneath the surface

In non-destructive imaging, the information about the spatial pattern of a samples interior has to be transferred to the sample surface by certain waves, e.g., thermal or ultrasound waves. At the sample surface these waves can be detected and the interior structure is reconstructed from the measured signals (Fig. 1). The amount of information about the interior of the sample, which can be gained from the detected waves on the sample surface, is essentially influenced by the propagation from its excitation to the surface. Scattering, dissipation, or diffusion causes entropy production which results in a loss of information for the propagating waves. A unifying framework for treating diverse diffusion-related periodic phenomena under the global mathematical label of diffusion-wave fields has been developed by Mandelis [1], such as thermal waves, charge-carrier-density waves, diffuse-photon-density waves, but also modulated eddy currents, neutron waves, or harmonic mass-transport diffusion waves.

Several attempts have been made to compensate for these diffusive or dissipative effects to get a higher resolution for the reconstructed images of the sample interior. In this work it is shown that thermodynamical fluctuations limit this compensation, and therefore, the spatial resolution for non-destructive imaging at a certain depth is also limited. This loss of information can be described by stochastic processes, e.g., for thermal diffusion with temperature as a random variable.

Thermography uses the time-dependent diffusion of heat (either pulsed or modulated periodically) which goes along with entropy production and a loss of information. Thermal waves are a good example as they exist only because of thermal diffusion. The amplitude of a thermal wave decreases by more than 500 times at a distance of just one wavelength [2].

Another example is ultrasound imaging, taking acoustic attenuation of the generated ultrasound wave into account. Here, the pressure of the acoustic wave is described by a stochastic process. As for any other dissipative process, the energy in attenuated acoustic waves is not lost but is transferred to heat, which can be described in thermodynamics by an entropy increase. This increase in entropy is equal to a loss of information, as defined by Shannon [3], and no algorithm can compensate for this loss of information. This is a limit given by the second law of thermodynamics.

The outstanding role of entropy and information in statistical mechanics was published in 1963 by Jaynes [4]. Already in 1957 he gave an information theoretical derivation of *equilibrium thermodynamics* showing that under all possible probability distributions with particular expectation values (equal to the macroscopic values like energy or pressure), the distribution which maximizes the Shannon information is realized in thermodynamics [5]. Jaynes explicitly showed for the canonical distribution, which is the thermal equilibrium distribution for a given mean value of the energy, that the Shannon or Gibbs entropy change is equal to the dissipated heat divided by the temperature, which is the entropy as defined in phenomenological thermodynamics [5, 6]. This “experimental entropy” in conventional thermodynamics is only defined for equilibrium states but by using the equality to Shannon information, Jaynes recognized that this “gives a generalized definition of entropy applicable to arbitrary nonequilibrium states, which still has the property that it can only increase in a reproducible experiment” [6].

In non-destructive imaging, the sample is not in equilibrium, e.g., for thermography a short pulse from a laser or a flash light induces a non-equilibrium state which in the end evolves into an equilibrium state. For states *near thermal equilibrium in the linear regime*, we use in the next section the theory of non-equilibrium thermodynamics presented by de Groot and Mazur [7]. In this regime microscopic time reversibility entails, as Onsager has first shown, a relation between fluctuation and dissipation, since one cannot distinguish between the average regression following an external perturbation or an equilibrium fluctuation [8]. This relation is known as the fluctuation–dissipation theorem and is due to Callen and Greene [9], Callen and Welton [10], and Greene and Callen [11]. It represents in fact a generalization of the famous Johnson [12]–Nyquist [13] formula in the theory of electric noise.

Over the last two decades, time reversibility of deterministic or stochastic dynamics has been shown to imply relations between fluctuation and dissipation in systems *far from equilibrium*, taking the form of a number of intriguing equalities, the fluctuation theorem [14, 15], the Jarzynski equality [16], and the Crooks relation [17]. The conceptual framework of “stochastic thermodynamics” relates applied or extracted work, exchanged heat, and changes in internal energy along a single fluctuating trajectory [18]. In an ensemble one gets probability distributions for these quantities, and since dissipated heat is typically associated with changes in entropy, one gets also a distribution for the entropy. In the next section a consequence of these equalities will be used: that thermodynamic entropy production is equal to the relative entropy, which is a quantitative measure of the information loss.

In this paper the measured variable, such as temperature, is treated as a time-dependent random variable with a mean value and a variance as a function of time. More about random variables and stochastic processes can be found, e.g., in the book about “Statistical Physics” from Honerkamp [19]. An introduction to stochastic processes on an elementary level has been published by Lemons [20], also containing “On the Theory of Brownian Motion” by Langevin [21]. An introduction to Markov processes on a slightly more advanced level is given by Gillespie [22]. To be able to use the results of some “model” stochastic processes given in the literature such as the Ornstein–Uhlenbeck process for a model of thermal waves, we have changed the initial conditions: instead of a defined initial value (with zero variance), we have taken the stochastic process at equilibrium before time zero. At time zero ($t = 0$) a certain perturbation has been applied to the process (e.g., a rapid change in velocity of a Brownian particle—called kicked Ornstein–Uhlenbeck process). Reconstruction of the size of this perturbation at time $t = 0$ from the measurement after a time $t$ shows how the information about the size of this kick at $t = 0$ gets lost with increasing time if diffusive or dissipative processes occur.

## Information Loss and Entropy Production

In this section we shall first assume that the time varying stochastic processes will have Gauss–Markov character. In doing so we do not wish to assume that all macroscopic processes considered belong to this specific class of processes. It may, however, be surmised that a number of real phenomena near equilibrium may, with a certain approximation, be adequately described by such Gauss–Markov processes [7]. At the end of this section we will see that the same results can be achieved for general processes from relations between fluctuation and dissipation in systems far from equilibrium, which have been derived in recent years.

The advantage of specifying more precisely the nature of the processes considered is that it enables us to discuss, on the level of the theory of random processes, the behavior of entropy production and of information loss. Following the theory of random fluctuations given, e.g., by de Groot and Mazur [7], we take as a starting point equations analogous to the Langevin equation used to describe the velocity of a Brownian particle:

1 $$\begin{aligned} \frac{\hbox {d}x}{\hbox {d}t}=-M\cdot x+\varepsilon (t) \end{aligned}$$

The components of the vector ***x*** are the random variables $x_i (i=1,2,\ldots ,n)$, which have at equilibrium a zero mean value. The matrix ***M*** of real phenomenological coefficients is independent of time $t$. The vector $\varepsilon (t)$ represents white noise, which is at different times uncorrelated. The distribution density of ***x*** turns out to be an $n$-dimensional Gaussian distribution:

2 $$\begin{aligned} f({\varvec{x}},t)=\frac{1}{\sqrt{(2\pi )^{n}\left| \Sigma \right| }}\;\hbox {e}^{-\frac{1}{2}({\varvec{x}}-\overline{{{\varvec{x}}}}(t))^{T}\Sigma ^{-1}({\varvec{x}}-\overline{{{\varvec{x}}}}(t))} \end{aligned}$$

with the mean value ${\overline{{\varvec{{x}}}}}(t)$ and the covariance matrix $\Sigma$, which is usually also a function of time $t.$ Using equilibrium ($\overline{{\varvec{{x}}}}_0 =0$) as initial conditions has a significant advantage compared to initial conditions used in the literature: it turns out that the covariance stays constant in time, while the mean value is a function of time. This facilitates the calculations of the entropy production and of the information loss due to the stochastic process. $| \Sigma |$ is the determinant of the covariance matrix.

If the initial mean value of ***x*** at time zero is given by $\overline{{{\varvec{x}}}}_0$, the mean value at a later time will be

3 $$\begin{aligned} \overline{{{\varvec{x}}}}(t)=\hbox {e}^{-{\varvec{M}}t}\cdot \overline{{{\varvec{x}}}}_0 \end{aligned}$$

By an adequate coordinate transformation of ***x***, the matrix ***M*** can be diagonalized: the eigenvalues of ***M*** are the elements of the diagonal matrix.

According to the second law of thermodynamics, the entropy of an adiabatically insulated system must increase monotonically until thermodynamic equilibrium is established within the system, where the entropy is set to zero. Then the entropy at a time $t$ is

4 $$\begin{aligned} S(t)=-k_\mathrm{B} \int {f({\varvec{x}},t)\ln \frac{f({\varvec{x}},t)}{f({\varvec{x}},t\rightarrow \infty )}\;} \hbox {d}{\varvec{x}}\equiv -k_\mathrm{B} D(\left. {f({\varvec{x}},t)} \right\| f({\varvec{x}},t\rightarrow \infty ))\qquad \end{aligned}$$

with the Boltzmann constant $k_\mathrm{B}$, ln is the logarithm to the base e, and $D$ is the relative entropy or Kullback–Leibler distance between the distribution at time $t$ and the distribution at equilibrium ($t$ at infinity). Stein’s lemma [23] gives a precise meaning to the relative entropy $D(f||g)$ between two distributions: if $n$ data from $g$ are given, the probability of guessing incorrectly that the data come from $f$ is bounded by e$^{-nD(f||g)}$, for $n$ large [24]. Therefore, the relative entropy is a quantitative measure of the information loss due to the stochastic process and is equal to the thermodynamic entropy $S(t)$.

For Gaussian distributions with a constant covariance matrix, the Kullback–Leibler distance results in

5 $$\begin{aligned} S(t)=-\frac{1}{2}k_\mathrm{B} \overline{{{\varvec{x}}}}(t)^{T}\Sigma ^{-1}\overline{{{\varvec{x}}}}(t) \end{aligned}$$

Before modeling heat diffusion as a Gauss–Markov process, we give a simple example: the Ornstein–Uhlenbeck process with only one component of ***x*** as a model, e.g., for the velocity of a Brownian particle.

### Kicked Ornstein–Uhlenbeck Process

If the random vector ***x*** in Eq. 1 has only one component, we get the Langevin equation

6 $$\begin{aligned} \frac{\hbox {d}v(t)}{\hbox {d}t}=-\gamma \cdot v(t)+\sigma \;\eta (t) \end{aligned}$$

which was used to describe the Brownian motion of a particle. The random variable $$v$$ is the particle velocity, $$-\gamma \cdot v$$ is the viscous drag, and $$\sigma $$ is the amplitude of the random fluctuations. The Langevin equation governs an Ornstein–Uhlenbeck process, after Ornstein and Uhlenbeck, who formalized the properties of this continuous Markov process [25]. Now we assume that initially we have thermal equilibrium with a zero mean velocity, and at time zero, the particle is kicked which causes an immediate change in the velocity of $$v_0 $$. Following Eq. 3, the mean value $$\overline{{v}}(t)$$ shows an exponential decay:

7 $$\begin{aligned} \overline{{v}}(t)=\hbox {e}^{-\gamma t}\cdot \overline{{v}}_0 \end{aligned}$$

**Fig. 2:**
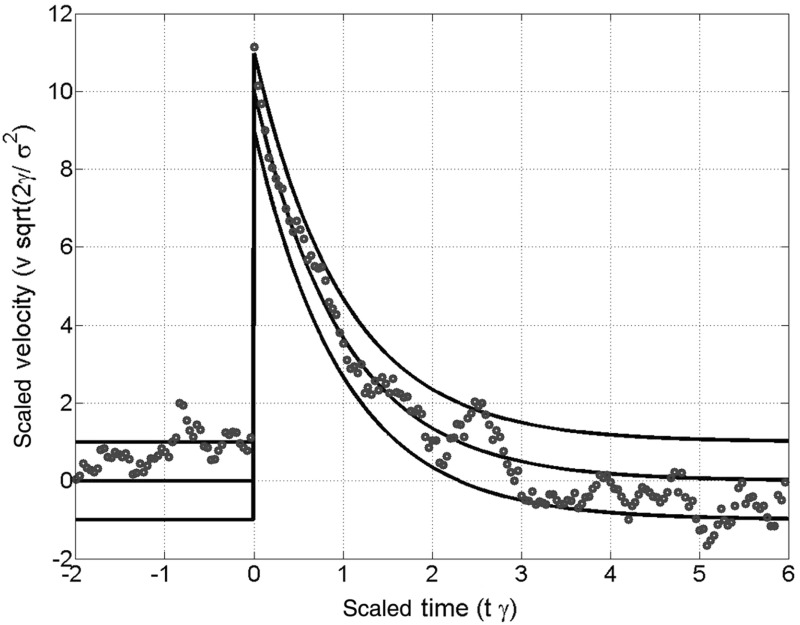
Points on a sample path of the normalized kicked Ornstein–Uhlenbeck process defined by the Langevin Eq. 6. The *solid lines* represent the mean, and mean $\pm$ standard deviation of the scaled velocity coordinate. At the time $t = 0$ a value of $v_{0} = 10$ has been added to the scaled velocity. After some time the information of the amplitude gets more and more lost due to the fluctuations

The variance of the velocity turns out to stay constant in time and is therefore equal to the equilibrium value $\hbox {Var}(v)={\sigma ^{2}}/{(2\gamma )}$ (e.g., [19]). In Fig. 2 the time and velocity are scaled to be dimensionless and the standard deviation (square root of the variance) of the velocity is normalized.

From Eq. 5 the information loss equal to the entropy production until the time $t$ after the kick is

8 $$\begin{aligned} \Delta S(t)=k_\mathrm{B} \frac{\gamma }{\sigma ^{2}}\overline{{v}}(t)^{2} \end{aligned}$$

On the other hand the entropy production known from thermodynamics is the dissipated energy $\Delta Q$, which is the kinetic energy of the Brownian particle of mass $m$, divided by the temperature $T$:

9 $$\begin{aligned} \Delta S(t)=\frac{\Delta Q}{T}=\frac{m\overline{{v}}(t)^{2}}{2T} \end{aligned}$$

The thermodynamic entropy production in Eq. 9 has to be equal to the loss of information in Eq. 8, and therefore we get for the variance of the velocity:

10 $$\begin{aligned} \frac{\sigma ^{2}}{2\gamma }=\frac{k_\mathrm{B} T}{m} \end{aligned}$$

Equation 10 states a connection between the strength of the fluctuations, given by $\sigma ^{2}$, and the strength of the dissipation $\gamma$. This is the fluctuation–dissipation theorem (FDT) in its simplest form for uncorrelated white noise, and this derivation of Eq. 10 shows the information theoretical background of the FDT.

Another simple model for a stochastic process is the stochastically damped kicked harmonic oscillator, which combines an oscillatory with a diffusive behavior and therefore is a good starting point to model attenuated acoustic waves. It can be treated similarly as the kicked Ornstein–Uhlenbeck process, and the equations of motion were solved already in 1943 by Chandrasekhar [26] for definite initial conditions $$x(0)$$ and $$p(0)$$. Again we have changed the initial conditions to an oscillator with zero mean values kicked by an initial momentum $$p_0 $$ at time zero. Further details are given in Sect. 5 in our book chapter on attenuated acoustic waves [27].

### Entropy Production for General Processes

In Sect. 2.1, the stochastic process was assumed to have Gauss–Markov character. Now we will see that the same results can be achieved for general processes from relations between fluctuation and dissipation in systems far from equilibrium, which have been derived the past years [24, 28, 29]: Jarzynski described a “forward” process starting from an equilibrium state at a temperature $T$, during which a system evolves in time as a control parameter $\lambda$ is varied from an initial value $A$ to a final value $B$. $W$ is the external work performed on the system during one realization of the process; $\Delta F=F_{B}-F_{A}$ is the free energy difference between two equilibrium states of the system, corresponding to $\lambda =A$ and $B$. The “reverse” process starts from an equilibrium state with $\lambda =B$ and evolves to $\lambda =A$. For the relative entropy $D$ between the forward and reverse processes, it was shown that

11 $$\begin{aligned} \Delta S=\frac{\left\langle W \right\rangle -\Delta F}{T}=k_\mathrm{B} D(\left. \rho \right\| \tilde{\rho }) \end{aligned}$$

with the average performed work $\left\langle W \right\rangle$ and the probability density $\rho$ in phase space for the forward process and $\tilde{\rho }$ for the reverse process. For a sudden change of the control parameter, which could be, e.g., the sudden heat pulse at a time $t=$0 for pulse thermography, this gives the same result as Eq. 4, but now not only for Gaussian distributions (details can be found in [29]).

As summarized in Sect. 2, the equity of thermodynamic entropy production and information loss—measured as the relative entropy $D$—was shown for general non-equilibrium processes. The underlying assumptions are that we start with an equilibrium process and the “disturbance” at time $t = 0$ has to be short, but it has not to be small. For thermography these conditions are fulfilled if the initial pulse is short compared to the time needed for the diffusion of heat. Therefore, the equity of thermodynamic entropy production and information loss will be applied to thermal diffusion after such a short pulse in the next section.

## Thermal Diffusion as a Stochastic Process

### Thermal Diffusion Equation in Real and Fourier Space

Thermal diffusion can be described by the differential equation (Fourier, 1823, or, e.g., Rosencwaig [2]):

12 $$\begin{aligned} \left( \Delta -\frac{1}{\alpha }\frac{\partial }{\partial t}\right) T({\varvec{r}},t)=-\frac{1}{\kappa }q({\varvec{r}},t) \end{aligned}$$

where $T$ is the temperature and $q$ is the thermal source volumetric density as a function of space ***r*** and time $t$. $\Delta$ is the Laplacian operator. $\alpha$ is the thermal diffusivity and $\kappa$ is the thermal conductivity, which are both material properties.

In the very well developed theory of heat transfer (e.g., by Carslaw and Jaeger [30]), usually a bilateral Fourier transform is applied to $T$ and $q$ in the diffusion equation:

13 $$\begin{aligned} g(t)&= \frac{1}{2\pi }\int \limits _{-\infty }^{+\infty } {G(} \omega )\exp (\hbox {i}\omega t) \hbox {d}\omega \nonumber \\ G(\omega )&= \int \limits _{-\infty }^{+\infty } {g(t)} \exp (-\hbox {i}\omega t) \hbox {d}t \end{aligned}$$

where $\hbox {i}=\sqrt{-1}$ and $\omega$ is the angular frequency. When Eq. 13 is applied to $T$ and $q$ in Eq. 12, one gets for the Fourier transformed $\theta$ and $Q$ the inhomogeneous form of the Helmholtz equation:

14 $$\begin{aligned} \left( \Delta -\frac{\hbox {i}\omega }{\alpha }\right) \theta \left( {\varvec{r}},\omega \right) =-\frac{1}{\kappa }Q\left( {\varvec{r}},\omega \right) \end{aligned}$$

This equation may be solved by the application of a bilateral Fourier transform over space, defined as

15 $$\begin{aligned} G({\varvec{r}})&= \frac{1}{(2\pi )^{3}}\int \!\!\!\int \!\!\!\int {\hat{{G}}({\varvec{k}})\exp (-\hbox {i}{\varvec{kr}})} \hbox {d}{\varvec{k}} \nonumber \\ \hat{{G}}({\varvec{k}})&= \int \!\!\!\int \!\!\!\int {G({\varvec{r}})} \exp (\hbox {i}{\varvec{kr}}) \hbox {d}{\varvec{r}} \end{aligned}$$

where ***k*** is the wave vector (see, e.g., Buckingham [31]). When Eq. 15 is applied to $\theta$ and $Q$ of Eq. 14, the Helmholtz equation reduces to

16 $$\begin{aligned} \hat{{\theta }}({\varvec{k}},\omega )=\frac{\alpha }{\kappa }\frac{\hat{{Q}}({\varvec{k}},\omega )}{\alpha \, {\varvec{k}}^{2}+\hbox {i}\omega } \end{aligned}$$

**Fig. 3:**
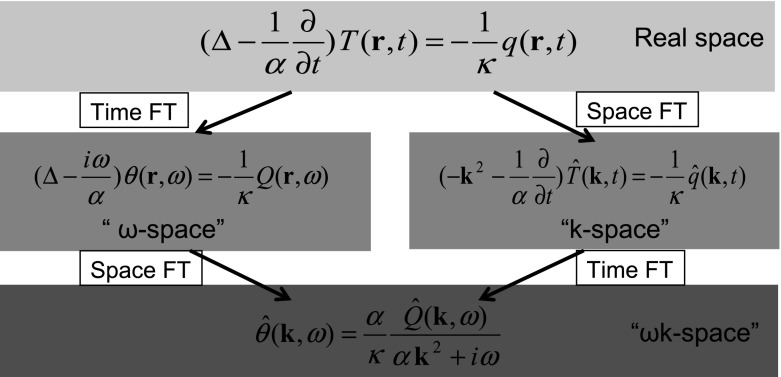
Diffusion Eq. 12 can be solved by a Fourier transform (FT) in time (Eq. 13) and a subsequent FT in space (Eq. 15). The same result in “$\omega k$-space” is gained if the space FT is performed first, and then the time FT

The same result can be achieved if first the spatial Fourier transform (Eq. 15) is applied to $T$ and $q$ in Eq. 12,

17 $$\begin{aligned} \left( -{\varvec{k}}^{2}-\frac{1}{\alpha }\frac{\partial }{\partial t}\right) \hat{{T}}({\varvec{k}},t)=-\frac{1}{\kappa }\hat{{q}}({\varvec{k}},t), \end{aligned}$$

and then the temporal Fourier transform (Eq. 13) is applied to Eq. 17, as shown in Fig. 3.

In the past, most publications have used the Helmholtz equation (“$\omega$-space” in the left of Fig. 3). For example, Mandelis has solved this equation in his book “Diffusion-Wave Fields” [1] in one, two, or three dimensions and for different boundary conditions (e.g., adiabatic boundary conditions where no heat can be “lost” at the sample boundary) by using Green functions. For a delta source in space and time and no boundaries, one gets in one dimension:

18 $$\begin{aligned} T(x,t)&= \frac{1}{4\pi }\int \limits _{-\infty }^{+\infty } {\exp (\hbox {i}\omega t)} \frac{1}{\sqrt{\hbox {i}\omega \alpha }}\exp \left( -\sqrt{\frac{\hbox {i}\omega }{\alpha } }\left| x \right| \right) \hbox {d}\omega \nonumber \\&= \frac{1}{2\pi }\int \limits _0^{+\infty } {\cos \left( \omega t-\sqrt{\frac{\omega }{2\alpha } }\left| x \right| -\frac{{\pi }}{4}\right) } \frac{1}{\sqrt{\omega \alpha }}\exp \left( -\sqrt{\frac{\omega }{2\alpha } }\left| x \right| \right) \hbox {d}\omega \nonumber \\&= \frac{1}{2\sqrt{\pi \,\alpha \, t}}\exp \left( -\frac{x^{2}}{4\alpha \,t}\right) \end{aligned}$$

The thermal wave is a superposition of waves with angular frequency $\omega$ and $\sqrt{\omega /{2\alpha }}$ as the wavenumber. At a distance $x$ the amplitude is damped by $\exp (-x\sqrt{\omega /{2\alpha }})$. Therefore, the damping factor after a distance of one wavelength is $\exp (-2{\pi })\approx 1/535$. This is the reason why thermal waves are a good example for “very dispersive” waves, as stated in the Introduction.

In “$k$-space” the thermal wave is a superposition of waves damped in time (see, e.g., Buckingham [31]). Now for a delta source in space and time and no boundaries, one gets in one dimension,

19 $$\begin{aligned} T(x,t)=\frac{1}{2{\pi }}\int \limits _{-\infty }^{+\infty } {\exp \left( -\hbox {i}kx\right) } \exp \left( -k^{2}\alpha \, t\right) \hbox {d}k=\frac{1}{2\sqrt{{\pi }\, \alpha \, t}}\exp \left( -\frac{x^{2}}{4\alpha t}\right) \qquad \end{aligned}$$

Of course, the result is the same as in Eq. 18. These two different representations of a damped wave correspond to a real frequency and a complex wave vector or vice versa. In the next section the exponential damping in time in Eq. 19 will be realized by an Ornstein–Uhlenbeck process, as described in Sect. 2.1.

### Thermal Diffusion Described by Ornstein–Uhlenbeck Processes

At time $t = 0$, an initial temperature distribution $T_0 ({\varvec{r}})$ is given on the whole sample volume $V$. The sample is thermally isolated, which results in adiabatic boundary conditions: the normal derivative of the temperature $T$ at the sample boundary vanishes at all times $t$. Therefore, after a long time $t$ a thermal equilibrium will be established with an equilibrium temperature $\overline{{T}}$.

For numerical calculations we use a discrete space $T_j =T({\varvec{r}}_j )$, where ${\varvec{r}}_j$ are $N$ points on a cubic lattice with a spacing of $\Delta r$ within the sample volume $V (j=1 ,{\ldots }N )$. At a time $t$ the temperature distribution $T_j (t)$ can be represented by the Fourier series [32] (including the time $t = 0$ with the known initial temperature distribution $T_0 ({\varvec{r}}))$,

20 $$\begin{aligned} T_j (t)=\;T({\varvec{r}}_j ,t)=\sum \limits _{k=0}^{N-1} {\hat{{T}}_k (t) } \phi _k ({\varvec{r}}_j )\quad \hbox {with}\;\phi _k ({\varvec{r}})=\hbox {e}^{2\pi i\varvec{\rho }_k \cdot {\varvec{r}}}\cdot I({\varvec{r}}). \end{aligned}$$

$I({\varvec{r}})$ is a support function which is one within the sample volume $V$ and zero outside. $\varvec{\rho }_k$’s are integer points on an infinite 3D lattice in $k$-space. The index $k = 0$ should correspond to $\rho _k =0$, and therefore, $\hat{{T}}_0 (t)$ is equal to the equilibrium temperature $\overline{{T}}$s which is constant in time. From the diffusion Eq. 12, we get

21 $$\begin{aligned} \hat{{T}}_k (t)=\hat{{T}}_k (0)\exp (-\gamma _k t)\quad \hbox {with}\;\gamma _k =4{\pi }^{2}\alpha \,\rho _k ^{2}. \end{aligned}$$

**Fig. 4:**
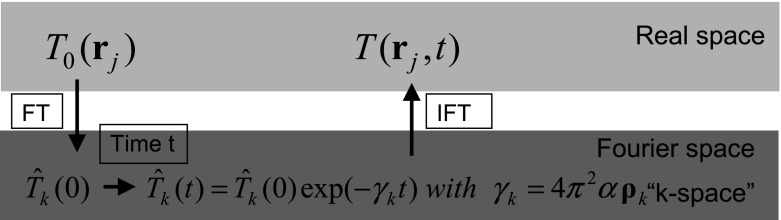
The initial temperature distribution $T_0 ({\varvec{r}})$ just after the laser pulse is Fourier transformed (FT). The time evolution of the Fourier series coefficients can be described similar to the mean value of an Ornstein–Uhlenbeck process (Eq. 7). The temperature distribution $T({\varvec{r}},t)$ after a time $t$ is then calculated by an inverse Fourier transform (IFT)

In Fourier space the time evolution is just an exponential decay in time (Fig. 4). Only for $k = 0$, where ${\rho }_k =0$, the Fourier coefficient is constant in time and is equal to the equilibrium temperature as stated above.

Now we assume that the temporal evaluation of the temperature $$T$$ is realized by a Gauss–Markov process. The coordinate transformation, which diagonalizes the matrix ***M*** from Eq. 3, is the Fourier transform Eq. 20. The elements of the diagonalized matrix ***M*** are the time constants $$\gamma _k $$ for the exponential decay of $$\overline{{\hat{{T}}}}_k (t)$$, which increase with a higher order of $$k$$ (quadratically with a length of $$\rho _{k})$$. To get the variance $$s_k^2 $$ the Gaussian distribution for $$T_k (t)$$, we use again the fact that the loss of information has to be equal to the thermodynamical entropy production. From Eq. 5 we get for the loss of information:

22 $$\begin{aligned} \Delta S(t)=\frac{1}{2}k_\mathrm{B} \sum _{k=1}^{N-1} {\frac{1}{s_k ^{2}}\overline{{\hat{{T}}}}_k (t)^{2}} \end{aligned}$$

The term with $k = 0$ is subtracted and therefore the sum starts with $k = 1$ to ensure that the information loss is zero for the equilibrium distribution. Compared to Eq. 5, the sign is changed as the entropy production is positive.

On the other hand one gets from thermodynamics for the entropy production of a discrete temperature distribution around the equilibrium value $\overline{{T}}$ in real space [7] and in Fourier space:

23 $$\begin{aligned} \Delta S(t)=\frac{C_V }{2\overline{{T}}^{2}}\sum _{j=1}^N {\delta V_j } (T_j -\overline{{T}})^{2}=\frac{C_V }{2\overline{{T}}^{2}}V\sum _{k=1}^{N-1} {\overline{{\hat{{T}}}}_k (t)^{2}} \end{aligned}$$

where $SV_{j}$ is the volume of volume element number $j$. Their sum is the sample volume $V$. $C_{V}$ is the heat capacity at a constant volume. Comparison with Eq. 22 shows that the variance in Fourier space is the same for all wavenumbers with index $k$:

24 $$\begin{aligned} s_k^2 =k_\mathrm{B} \frac{\overline{{T}}^{2}}{C_V V} \end{aligned}$$

For each wave number index $k$, $\gamma _{k }$ in Eq. 21 gives the strength of dissipation and $s_k \sqrt{2\gamma _k }$ gives the amplitude of the random fluctuations for the Ornstein–Uhlenbeck process for $\hat{{T}}_k (t)$. The stochastic differential equation according to Eq. 6 is

25 $$\begin{aligned} \frac{\hbox {d}\hat{{T}}_k (t)}{\hbox {d}t}=-\gamma _k \cdot \hat{{T}}_k (t)+s_k \sqrt{2\gamma _k }\;\eta (t);\quad k=1,{\ldots },N-1 \end{aligned}$$

**Fig. 5:**
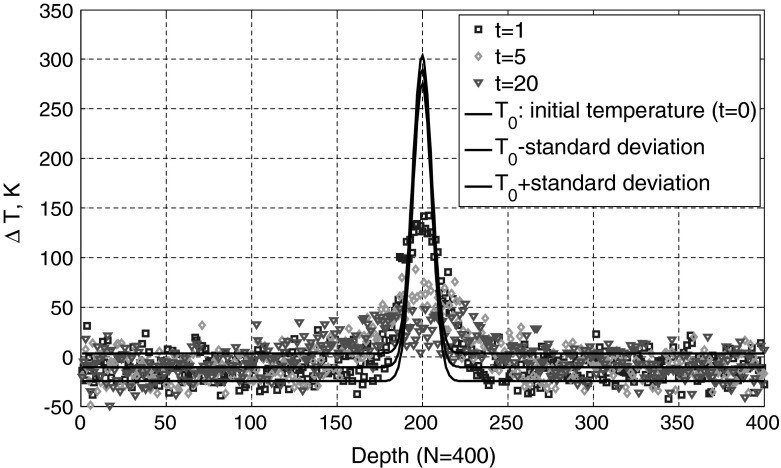
Deviation from equilibrium temperature $\Delta T=T-\overline{{T}}$ in real space as a funtion of time. The initial temperature distribution is Gaussian-shaped with a maximum of 600 K at a depth of 200 and the full width at 1/e of the maximum is 16 arb. units. The minimum temperature is 300 K. The standard deviation in real space is constant in depth and time

**Fig. 6:**
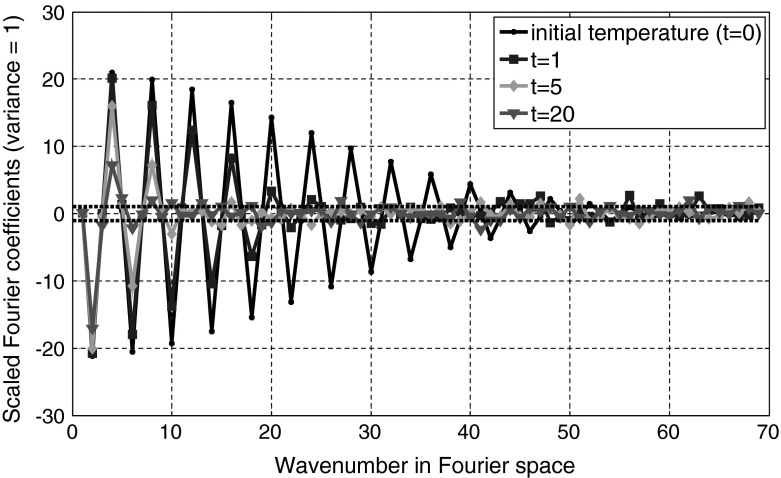
Fourier coefficients as a function of time. Coefficients with a higher wavenumber show a rapid decay with time according to Eq. 21. The Fourier coefficients are scaled to have a variance of one (independent of wavenumber)

Figures 5 and 6 show a one-dimensional example for $$N=400$$. The initial temperature distribution is Gaussian-shaped with a maximum of 600 K at a depth of 200 and the full width at 1/e of the maximum is 16 arb. units. The minimum temperature is 300 K. The deviation from equilibrium temperature $$\Delta T=T-\overline{{T}}$$ is shown in black in Fig. 5 in real space and in Fig. 6 in Fourier space (“$$k$$-space”). The thermal diffusivity is chosen as 50. The temperature profiles at the 400 discrete points are calculated by 400 independent Ornstein–Uhlenbeck processes for $$\hat{{T}}_k (t)$$ at a time $$t = 1$$, $$t = 5$$, and $$t = 20$$. In Fourier space higher orders of $$k$$ show a rapid decrease. The Fourier coefficients are scaled to give a variance $$s_k^2 =1$$. The standard deviation in real space is 14.12 K and is constant in depth and time, as the initial state is the temperature distribution of the equilibrium state increased by $$T_0 $$ (analog to the kicked Ornstein–Uhlenbeck process). The standard deviation is selected rather high for this simulation to show clearly the influence of fluctuations.

After a long time the mean value of all Fourier coefficients will vanish. Only the Fourier coefficient for $$k = 0$$ is constant and equal to the equilibrium temperature. The entropy from Eq. 22 goes to zero, and all the information about the shape of the initial temperature profile is lost. For earlier times the temperature profile is broadened (Fig. 5) but the initial temperature can be reconstructed to a certain extent, which depends on the fluctuations $$s_k \sqrt{2\gamma _k }$$. How to get the best reconstruction of the initial temperature profile from a “measured” temperature distribution at a later time $$t $$ will be the topic of the next section.

### Time Reversal and Regularization Methods

In the previous section the initial temperature distribution $T_0 ({\varvec{r}})$ was given and the temperature distribution $T({\varvec{r}},t)$ after a time $t$ was calculated (Fig. 4). Now we want to reconstruct the initial temperature distribution from the measured temperature distribution at a time $t$ (inverse problem). Without fluctuations ($s_k =0$), this reconstruction in $k$-space would be (from Eq. 21)

26 $$\begin{aligned} \hat{{T}}_k (0)=\hat{{T}}_k (t)\exp (+\gamma _k t)\quad \hbox {with}\;\gamma _k =4{\pi }^{2}\alpha {\rho }_k ^{2}. \end{aligned}$$

These exponential time evolution factors can reach high values for increasing length of wave vectors $\rho _k$. Then even small fluctuations are “blown up” exponentially and the reconstruction error is big. Therefore, regularization methods have to be used for this inverse problem, like truncated singular value decomposition (SVD) or Tikhonov regularization [33]. For truncated SVD only the first $i$ wavenumbers are taken and the other wavenumbers for which the exponential factors produce high errors are set to zero:

27 $$\begin{aligned} \hat{{T}}_k (0)=\left\{ \begin{array}{ll} \hat{{T}}_k (t)\exp (+\gamma _k t), &{} \hbox {for}\;k\le i \\ 0 &{} \hbox {otherwise} \\ \end{array} \right\} . \end{aligned}$$

Tikhonov regularization looks for a $\hat{{T}}(0)$ which fulfills the linear vector equation $\hat{{T}}(t)=\hat{{T}}(0)\exp (-\varvec{\gamma }t)$ as good as possible, but is also a smooth solution, minimizing ${\hat{T}}(0)^{2}.$ The regularization parameter $\lambda$ gives the tradeoff between these two requirements: $\min \{(({\hat{T}}(t)-{\hat{T}}(0)\exp (-{\gamma }t))^{2}+\lambda {\hat{T}}(0)^{2}\}$. Differentiating with respect to $\hat{{T}}_k (0)$ and setting the derivative equal to zero results in

28 $$\begin{aligned} \hat{{T}}_k (0)=\frac{\exp (-\gamma _k t)}{\exp (-2\gamma _k t)+\lambda }\hat{{T}}_k (t) \end{aligned}$$

**Fig. 7:**
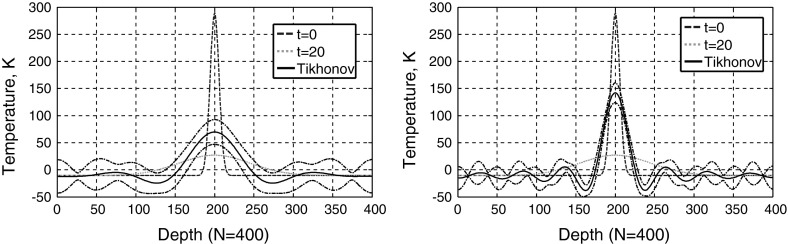
Tikhonov reconstruction of the initial temperature and its standard deviation for a low signal-to-noise-ratio (SNR) from the data shown in Fig. 5 (*left*) and a SNR multiplied by 10$^{5}$ (*right*). The width of the reconstructed peak is smaller for the higher SNR and therefore enables a better spatial resolution

Figure 7, left, shows the Tikhonov reconstruction of the initial temperature distribution and its standard deviation from the “measured” data shown in Fig. 5 at a time $t = 20$ with a variance in Fourier space ($k$-space) of $s_k^2 =1$ (Fig. 6). Figure 7, right, shows the reconstruction for the signal-to-noise ratio (SNR) multiplied by $10^{5} (s_k^2 =10^{-10})$. The width of the reconstructed peak is smaller for the higher SNR and therefore enables a better spatial resolution. The spatial resolution is defined as the minimal distance of two peaks, which can still be reconstructed as two individual peaks. This is equal to the width of the peak, if the initial temperature peak at $t = 0$ is a sharp peak like a delta source in space and time. The analytic expression is shown in Eq. 19 and shows a width (standard deviation) of $\sqrt{ 2\alpha \, t}$. In $k$-space $\hat{{T}}(k,t)=\exp (-k^{2}\alpha \, t)$ and the width is the inverse $\frac{1}{\sqrt{2 \alpha \, t}}$.

In $k$-space SVD reconstruction (Eq. 27), and in a good approximation for higher signal-to-noise ratios (SNRs), also Thikhonov reconstruction (Eq. 28) gives a rectangular function up to $k_{i}$ with $\exp (k_i ^{2}\alpha t) =\hbox {SNR}$. After Fourier transformation this gives a sinc function in real space for the reconstructed initial temperature profile (Fig. 7):

29 $$\begin{aligned} T_{\hbox {reconstruction}} (x,t=0)=\sqrt{\frac{2}{{\pi }}}\frac{\sin (k_i x)}{x}\quad \hbox {with}\quad k_i =\sqrt{\frac{\ln (\hbox {SNR})}{\alpha \, t}} \end{aligned}$$

**Fig. 8:**
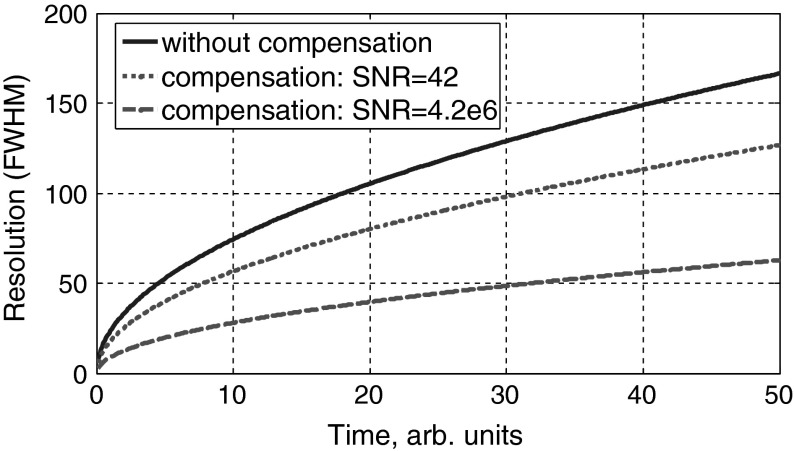
Possible spatial resolution (FWHM) from a measured temperature profile after a certain time without compensation (*solid line*) and with compensation for a small signal-to-noise ratio (SNR) from the data shown in Fig. 5 (*dotted line*) and a SNR of $4.2\times 10^{6}$ (*dashed line*)

In Fig. 8 the spatial resolution (defined as the width between the two inflection points of the central peak of the sinc function) for a reconstructed initial temperature profile from the measured temperature after a certain time is shown. This width can be approximated for higher SNRs by the distance between the zero points of the reconstructed temperature profile:

30 $$\begin{aligned} \hbox {Resolution}=\frac{2{\pi }}{k_i } =2{\pi }\sqrt{ \alpha \, t/\ln (\hbox {SNR})}. \end{aligned}$$

## Discussion, Conclusions, and Outlook

In Sect. 3 the whole one-dimensional temperature distribution $T(x,t)$ was detected at a certain time $t$ after a short generation pulse. From this data the initial temperature distribution $T(x,t=0)$ was reconstructed. For this situation we could give the limit of spatial resolution as a function of the signal-to-noise ratio (SNR) and time $t$.

In non-destructive imaging as sketched in Fig. 1, the temperature $T(x = x_\mathrm{S},t)$ is measured at the surface $x=x_\mathrm{S }$ of the sample as a function of time. In that case the limit of spatial resolution can be given as a function of SNR and depth. For this ongoing work the heat diffusion is modeled as a stochastic process in “$\omega$-space” (Fig. 3) instead of “$k$-space,” as was done in Sect. 3 (Fig. 4).

Future work should describe the limit of spatial resolution for different generation—not only for short pulses as in this contribution. This could, for example, describe the difference of the spatial resolution for lock-in thermography and pulse thermography. It is also planned to extend this work to two- or three-dimensional problems.

A similar description with pressure as a random variable can be used to describe the possible compensation of ultrasound attenuation in photoacoustic imaging [27].
